# Aberrant sialylation in cancer: From molecular mechanisms to potential therapeutics

**DOI:** 10.1016/j.gendis.2026.102080

**Published:** 2026-02-14

**Authors:** Ran Kong, Cong Wang, Yu Zhang, Guangcai Zhong, Jiarui Liu, Xiangxiang Zhou

**Affiliations:** Department of Hematology, Shandong Provincial Hospital Affiliated to Shandong First Medical University, Jinan, Shandong 250021, China

**Keywords:** Neuraminidases, Sialylation, Sialyltransferases, Targeted therapy, Tumorigenesis

## Abstract

Sialylation, a dynamic post-translational modification catalyzed by sialyltransferases and counterbalanced by neuraminidases, entails the attachment of sialic acids to the terminal residues of glycoproteins and glycolipids. This modification profoundly influences diverse biological processes, including early embryogenesis, neurodevelopment, maintenance of stem cell pluripotency, and oncogenic transformation. In cancer, aberrant sialylation manifests as altered linkage patterns and dysregulated expression of sialylated glycans, which directly drive malignant behaviors such as uncontrolled proliferation, enhanced adhesion and invasion, immune evasion, and therapy resistance. Deciphering the underlying molecular mechanisms is therefore crucial for advancing our understanding of tumor biology. In this review, we systematically summarize recent advances in the study of sialylation in cancer, with a focus on the biological functions of distinct sialyltransferases and neuraminidases. We further discuss the diagnostic, prognostic, and therapeutic implications of targeting sialylation, highlighting its emerging potential as a promising avenue for cancer treatment.

## Introduction

**Figure 1:**
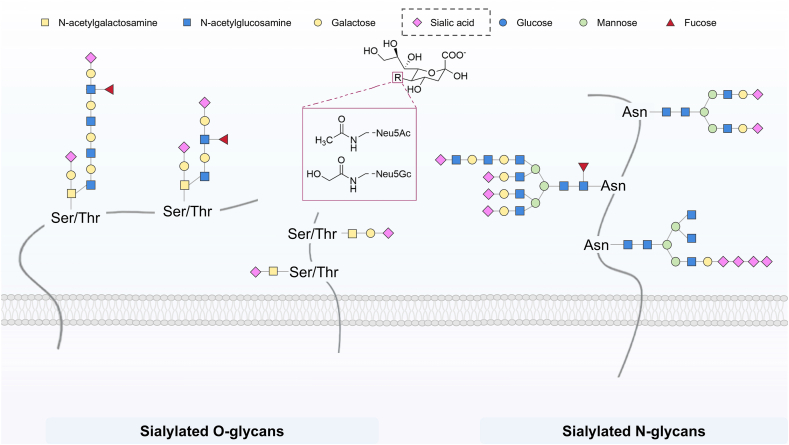
Sialylation on N- and O-linked glycans. Sialic acids are typically found at the terminal positions of both N- and O-linked glycans. O-linked glycosylation is initiated by the attachment of N-acetylgalactosamine (GalNAc) to serine (Ser) or threonine (Thr) residues. In contrast, N-linked glycosylation begins with the attachment of N-acetylglucosamine (GlcNAc) to asparagine (Asn) within the conserved sequon Asn-X-Ser/Thr and features a characteristic core pentasaccharide structure. A hallmark of cancer cells is hypersialylation. This figure illustrates common structural patterns of hypersialylation occurring on both N- and O-linked glycans. Neu5Ac, N-acetylneuraminic acid. Neu5Gc, N-glycolylneuraminic acid.

Glycosylation, a fundamental and highly dynamic post-translational modification, operates under both physiological and pathological conditions.^1^ This process entails the enzymatic transfer of glycans to proteins through glycosyltransferase-mediated formation of glycosidic bonds with specific amino acid residues.^2^ Based on the nature of the linkage between sugar chains and proteins, glycosylation is primarily categorized into N-glycosylation and O-glycosylation.^3^ Sialic acids, typically located at the terminal positions of both N- and O-linked glycans, constitute key monosaccharides that confer functional and structural diversity to glycoconjugates 4, 5, 6 (Fig. 1). Recent discoveries have revealed that sialylated N-glycans can also be conjugated to small RNAs, forming glycoRNAs that are modified across several cell types and present at the cell surface.^7^^,^^8^

**Figure 2:**
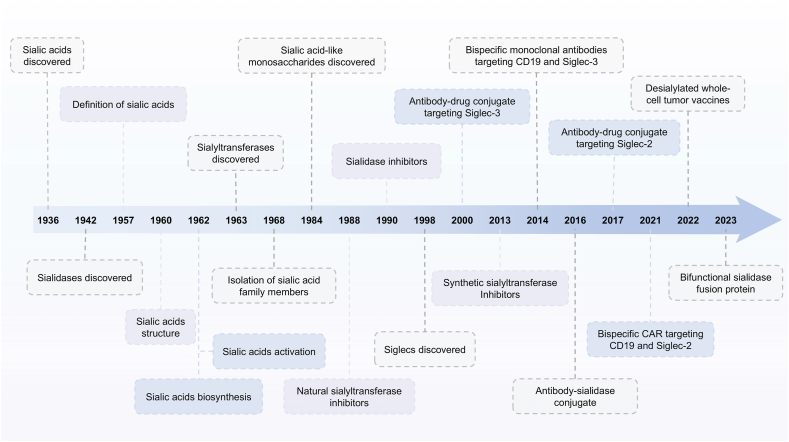
Timeline of sialylation development. The chronological development and ongoing exploration of sialylation reveal its significance.

**Figure 3:**
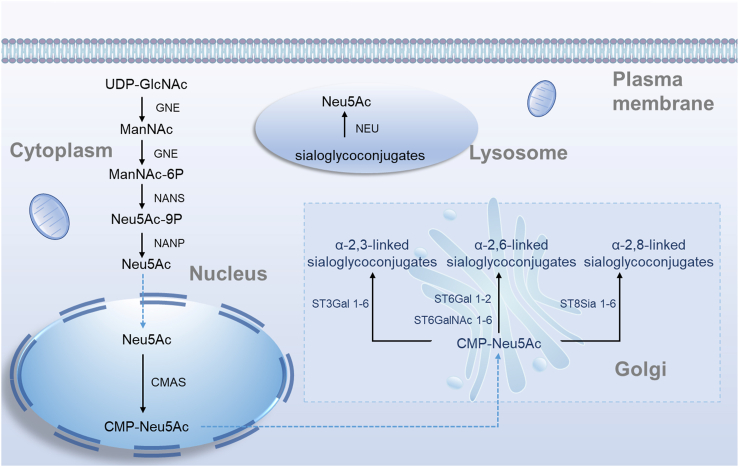
The biosynthesis pathway of sialylation. The metabolic pathways of sialylation involve a coordinated effort of specific enzymes that facilitate the biosynthesis, activation, and transfer of sialic acids to glycoconjugates, in addition to their removal and degradation. These enzymes work in concert to maintain the balance of sialic acid levels, which is crucial for various cellular functions. GNE, glucosamine (UDP-N-acetyl)-2-epimerase/N-acetylmannosamine kinase; NANS, N-acetylneuraminic acid synthase; NANP, N-acetylneuraminic acid phosphatase; CMAS, CMP-sialic acid synthetase.

Since its discovery in 1936, sialic acid biology has evolved into a rapidly expanding field (Fig. 2). N-acetylneuraminic acid (Neu5Ac) represents the predominant sialic acid species in humans.^4^^,^^9^ Although humans lack the enzymatic pathway to biosynthesize N-glycolylneuraminic acid (Neu5Gc), trace amounts of Neu5Gc are nevertheless detected in human tissues owing to dietary incorporation from red meat and dairy products.^10^, ^11^, ^12^ The structural diversity of sialylated glycans underlies their involvement in a variety of biological processes, ranging from embryonic and neural development to somatic cell reprogramming, stem cell pluripotency, and tumor progression.^13^ Sialylation is tightly regulated by two enzyme systems: sialyltransferases, which catalyze the transfer of sialic acids to glycoproteins and glycolipids, and neuraminidases (NEUs), which remove terminal sialic acids (Fig. 3). Mammals express four neuraminidases (NEU1–4) and four major sialyltransferase families, ST3Gal, ST6Gal, ST6GalNAc, and ST8Sia, each defined by their glycosidic linkage specificity.^14^^,^^15^ Dysregulation of these enzymes, primarily through the up-regulation of sialyltransferases, enhances sialylation and promotes glycosylation alterations that drive cancer development and progression.

Sialic acid-binding immunoglobulin-like lectins (Siglecs) constitute a family of receptors predominantly expressed on immune cells that recognize sialoglycans as ligands. To date, 15 Siglec receptors have been identified in humans.^16^ Most possess inhibitory intracellular domains that, upon ligand engagement, trigger downstream signaling cascades leading to immune suppression.^17^ Within the tumor microenvironment, hypersialylation on cancer cells can engage Siglecs on immune cells, establishing an immunosuppressive niche that facilitates tumor immune evasion.^17^^,^^18^

Given its multifaceted role in tumorigenesis and disease progression, dissecting the mechanisms of aberrant sialylation is essential for understanding cancer biology and identifying novel therapeutic avenues. In this review, we summarize recent advances in the study of sialylation in cancer, introduce several detection technologies, and highlight the emerging potential of targeting sialylation for cancer therapy.

## Modification of sialylation

### Sialyltransferase-mediated sialylation

Sialyltransferases, a specialized subclass of glycosyltransferases, are key enzymes that determine the extent of sialylation. They play indispensable roles in numerous physiological and pathological processes, including cancer, infectious diseases, and inflammation.^19^ These enzymes catalyze the formation of different glycosidic linkages, including α2,3-, α2,6-, or α2,8-linkage, using cytidine monophosphate N-acetylneuraminic acid (CMP-Neu5Ac) as the sugar donor.^20^ According to the type of glycosidic linkage formed, mammalian sialyltransferases are classified into four major families: ST3Gal, ST6Gal, ST6GalNAc, and ST8Sia.^21^^,^^22^ Aberrant up-regulation of sialyltransferase expression or activity frequently results in hypersialylation of the cell surface, a hallmark of malignant transformation and immune escape. Increasing evidence indicates that the expression of sialyltransferases correlates with disease aggressiveness and poor clinical outcomes. Consequently, targeting sialyltransferases has emerged as a promising therapeutic strategy for restoring sialylation homeostasis and attenuating disease progression.

### Neuraminidase-mediated desialylation

NEUs, also known as sialidases, catalyze the removal of terminal sialic acids from glycoconjugates and thereby modulate a broad spectrum of cellular processes. In mammals, four distinct NEU enzymes, NEU1, NEU2, NEU3, and NEU4, have been identified, each exhibiting unique subcellular localization and functional specificity.^21^^,^^22^ NEU1, predominantly localized within lysosomes, regulates multiple cellular functions, including immune responses, exocytosis, and elastic fiber assembly.^23^^,^^24^ NEU2, distributed in the cytoplasm and plasma membrane, participates in myoblast differentiation and neuronal development.^23^ NEU3 is primarily associated with the plasma membrane, whereas NEU4 localizes to several intracellular compartments such as lysosomes, mitochondria, and the endoplasmic reticulum.^24^ Together, NEU3 and NEU4 orchestrate key cellular processes, including cell adhesion, apoptosis, and neural differentiation.^24^^,^^25^ Beyond their physiological roles, alterations in NEU expression or activity have been implicated in various pathological states. Dysregulated desialylation contributes to neurodegenerative diseases, tumorigenesis, and infectious pathologies by disrupting sialic acid-dependent signaling pathways. Consequently, the pharmacological modulation of NEUs represents a promising therapeutic strategy for restoring glycan homeostasis and ameliorating disease progression.

## Sialylation in solid malignancies

**Figure 4:**
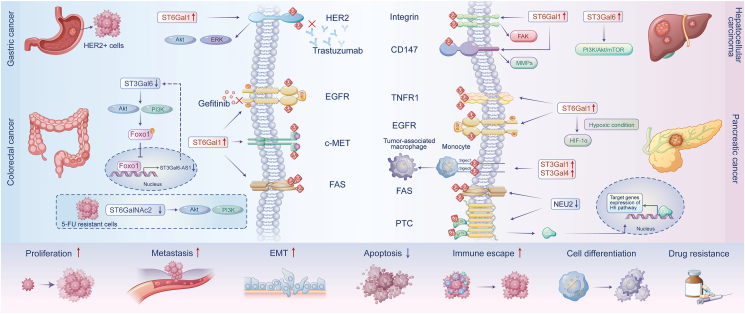
Alterations in the levels of sialylation-related enzymes under various conditions and their impact on tumorigenesis within the digestive system.

**Figure 5:**
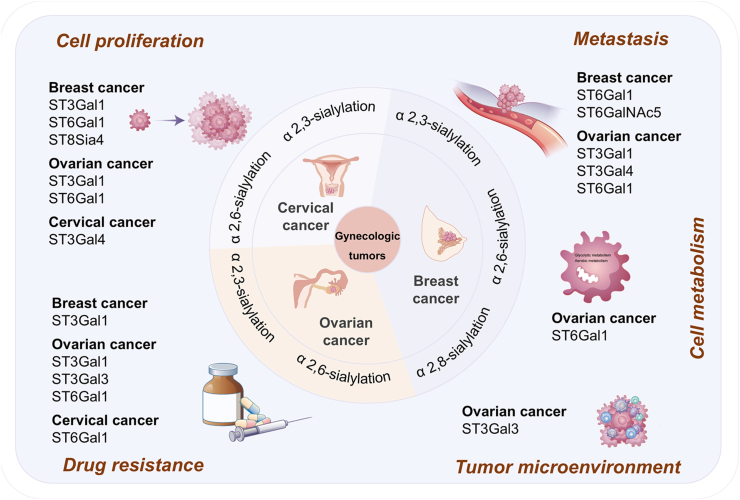
Variations of enzymes implicated in sialylation and their contributions to the oncogenic progression of gynecologic tumors.

A growing body of evidence has established altered sialylation as a pivotal event in the initiation and progression of numerous solid tumors, including those affecting the digestive system (Fig. 4), urinary system, and reproductive system (Fig. 5), and others.^1^^,^^26^, ^27^, ^28^, ^29^, ^30^ Understanding the molecular mechanisms by which sialylation influences cancer biology is therefore a central focus of contemporary tumor research, offering potential insights into novel therapeutic strategies.

### Gastric cancer

Gastric cancer ranks as the fifth leading cause of cancer-related morbidity and mortality worldwide, with limited therapeutic options available for improving patient prognosis.^31^, ^32^, ^33^, ^34^ Emerging evidence highlights the critical role of altered sialylation in gastric cancer progression, particularly through the up-regulation of sialyltransferases such as ST3Gal4. Gastric cancer tissues exhibit significantly higher expression of ST3Gal4 compared with normal tissues, with elevated α2,3-sialylation levels correlating strongly with increased cell invasion and metastasis of gastric cancer cells.^35^ Aberrant sialylation also contributes to the development of chemotherapy resistance in gastric cancer. Trastuzumab, a key therapeutic agent for human epidermal growth factor receptor 2 (HER2)-positive gastric cancer, primarily functions by inhibiting HER2 dimerization and promoting receptor internalization, followed by intracellular degradation, which ultimately leads to cell cycle arrest and suppression of tumor cell proliferation.^36^ However, a proportion of advanced HER2-positive gastric cancer patients acquire resistance to trastuzumab over time.^37^ Recent studies have linked this resistance to aberrant sialylation, particularly via the ST6Gal1 enzyme.^38^ Specifically, overexpression of ST6Gal1 promotes HER2 α2,6-sialylation via the phosphoinositide 3-kinase (PI3K)/protein kinase B (Akt) and extracellular signal-regulated kinase (ERK) pathways, inhibiting chemotherapy-induced apoptosis.^38^ Knockout of ST6Gal1 has been shown to prolong the half-life of HER2 and increase gastric cancer cell susceptibility to trastuzumab-mediated cytotoxicity.^36^^,^^38^ Ongoing research continues to reveal new insights into the role of sialylation in gastric cancer, offering the potential for the identification of novel therapeutic targets and the development of innovative treatment strategies.

### Colorectal cancer

Colorectal cancer (CRC) remains a major health challenge, with rising incidence and mortality rates, particularly in China.^39^^,^^40^ The pathogenesis of CRC is complex, involving multiple genetic and epigenetic alterations. A key player in CRC progression is altered sialylation, with the dynamic expression of ST6Gal1 observed across different stages of the disease. Specifically, ST6Gal1 levels are significantly higher in stages I and II compared with stages III and IV, suggesting that sialylation plays an important role in the progression of CRC.^41^

Due to frequent tumor recurrence and metastasis, CRC remains a leading cause of cancer-related mortality.^42^^,^^43^ Identifying key metastatic factors and elucidating the underlying molecular mechanisms are essential for improving patient prognosis. Aberrant sialylation has emerged as a significant driver of dysregulated signaling pathways that facilitate metastatic spread and sustain cancer cell proliferation in CRC. Sialylated immunoglobulin G (SIA-IgG), a glycosylated protein, enhances c-Myc protein levels by reducing its ubiquitination, thus promoting CRC cell migration, invasion, and liver metastasis. An anti-sialylated IgG antibody has been shown to effectively inhibit liver metastasis in mouse models, suggesting its potential as a therapeutic agent.^44^ In addition, reduced expression of ST3Gal6 and ST3Gal6 antisense 1 (ST3Gal6-AS1) has been observed in CRC tissues, along with a negative correlation between ST3Gal6-AS1 expression and distant metastasis.^45^ The ST3Gal6-AS1/ST3Gal6 axis induces α2,3-sialylation and suppresses the PI3K/Akt signaling pathway. Foxo1, a downstream effector of PI3K/Akt, regulates ST3Gal6-AS1 expression, creating a positive feedback loop.^46^, ^47^, ^48^ This axis represents a critical target for therapeutic intervention. Aberrant sialylation also contributes to the inhibition of apoptosis and sustains cancer cell proliferation in CRC cells. For instance, α2,6-sialylation of β1 integrin prevents Galectin-3-induced apoptosis, while sialylation of the FAS receptor by ST6Gal1 protects cells from FAS-mediated apoptosis by disrupting the interaction between FAS-associated death domain protein (FADD) and the cytoplasmic domain of FAS and blocking activated FAS receptor internalization.^49^^,^^50^ These findings provide important clues for a deeper understanding of the development of CRC and provide guidance for future research and clinical practice.

**Figure 6:**
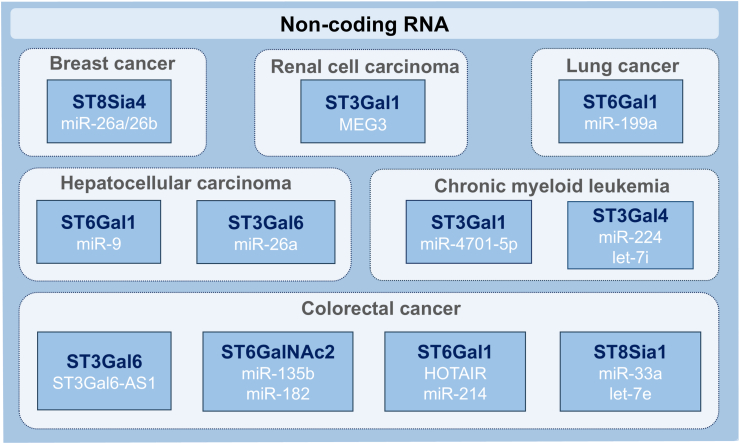
Sialylation-related enzymes regulated by non-coding RNAs in different human malignancies.

The standard management of CRC primarily involves surgical resection, often complemented by adjuvant chemotherapy.^51^ Sialylation is also closely linked to chemotherapy resistance in CRC. ST8Sia1, a sialyltransferase highly expressed in drug-resistant CRC cell lines and tissues, is negatively correlated with microRNA-33a (miR-33a) and hsa-let-7e (let-7e) expression (Fig. 6).^52^ Suppression of these microRNAs leads to increased ST8Sia1 expression, enhancing chemoresistance and promoting CRC cell proliferation, invasion, and angiogenesis.^52^ Conversely, ST8Sia1 knockout reduces tumor progression, making the miR-33a/let-7e/ST8Sia1 axis a potential therapeutic target for overcoming chemoresistance.^52^ Furthermore, the up-regulation of miR-135b or miR-182 in CRC cells promotes drug resistance and cell proliferation by down-regulating ST6GalNAc2, a key sialyltransferase, through the PI3K/Akt pathway (Fig. 6).^53^^,^^54^ Up-regulation of ST6Gal1 is a marker of poor prognosis in CRC, particularly in rectal cancer, where it is linked to treatment resistance. The oncogenic lncRNA HOX transcript antisense RNA (HOTAIR) mediates resistance to 5-fluorouracil in CRC by regulating ST6Gal1 expression.^55^ Regulation of miR-214 also results in the altered ST6Gal1 levels (Fig. 6). Increased ST6Gal1 promotes the sialylation of cellular-mesenchymal to epithelial transition factor (c-MET), which activates the JAK2/STAT3 pathway, further driving CRC progression.^55^^,^^56^ Knockdown of ST6Gal1 eliminates resistance to chemoradiation in CRC, highlighting the therapeutic potential of targeting sialylation to overcome chemoresistance.^57^

Immunotherapies and targeted therapies have emerged as promising treatment options for patients with unresectable or metastatic CRC. However, the development of resistance remains a critical clinical challenge.^51^^,^^58^ The small molecule inhibitor gefitinib, which targets epidermal growth factor receptor (EGFR), impedes CRC cell proliferation by blocking EGFR-mediated signaling. ST6Gal1 enhances the sialylation of both wild-type and mutant EGFR, contributing to gefitinib resistance.^59^ Sialylation significantly modulates the anti-tumor immune response. While the removal of sialic acid enhances anti-tumor immunity, this effect does not respond to further augmentation with anti-programmed death-ligand 1 (PD-L1) checkpoint blockade.^60^ However, knockdown of ST6Gal1 reduces the α2,6-sialylation of PD-L1, leading to its destabilization, ubiquitination, and subsequent degradation. ST6Gal1 knockdown, in combination with anti-PD-L1 therapy, enhances tumor response *in vivo*, highlighting ST6Gal1 as a potential target to mitigate drug resistance in immunotherapy.^61^ In conclusion, targeting sialylation may be a potential treatment option for patients with CRC.

### Hepatocellular carcinoma

Hepatocellular carcinoma (HCC) is the third leading cause of cancer-related death worldwide and the sixth most common cancer overall.^62^^,^^63^ HCC is often diagnosed at an advanced stage due to the absence of early symptoms. For patients with advanced HCC, systemic therapy is the primary treatment strategy. However, existing treatment options have limitations in terms of efficacy and applicability, underscoring the urgent need to develop novel therapeutic strategies and identify new molecular targets.^64^^,^^65^ Novel therapies targeting sialyltransferases may provide promising perspectives for the future. ST3Gal6 expression is up-regulated in HCC tissues and cell lines. miR-26a inhibits the activation of the PI3K/Akt/mTOR pathway by directly targeting ST3Gal6, significantly reducing tumor growth (Fig. 6).^66^ Additionally, in the T cell-tumor cell co-culture assay, up-regulation of ST6Gal1 suppresses the proliferation of T cells, enhances the secretion of interleukin-10 (IL-10) and transforming growth factor beta 1 (TGF-β1), and reduces the secretion of interferon gamma (IFN-γ) and tumor necrosis factor-alpha (TNF-α), thus promoting immune escape via the CD147/matrix metalloproteinases (MMPs) signaling pathway.^67^ miR-9 could partially restrain the metastatic potential by down-regulating ST6Gal1 expression in HCC cells (Fig. 6). By inhibiting α2,6-sialylation, miR-9 reduces cell migration and invasion, thereby slowing tumor progression in mouse hepatoma cells.^68^ While the majority of studies support that α2,6-sialylation contributes to tumor progression, a recent study indicates that ST6Gal1 may play a tumor-suppressive role in HCC. Zhou et al observed that metastatic cell lines express low or undetectable levels of ST6Gal1 compared with non-metastatic or low-metastatic cell lines. Moreover, ST6Gal1 expression negatively correlates with the metastatic potential and poor pathological features of HCC.^69^ In summary, sialylation plays a key role in the complex regulatory network of HCC. While current research has provided preliminary insights into its therapeutic potential, further in-depth investigation of sialyltransferases will be crucial for future therapeutic advancements.

### Pancreatic cancer

Acinar-to-ductal metaplasia is an early event in the development of pancreatic neoplasia. Sialylation mediated by ST6Gal1 could increase EGFR activation, thereby promoting acinar-to-ductal metaplasia progression.^70^ Cells undergoing acinar-to-ductal metaplasia in response to oncogenic signaling serve as precursors for pancreatic intraepithelial neoplasia lesions, which can progress to pancreatic ductal adenocarcinoma (PDAC).^71^ Metastasis is a hallmark of PDAC, with over 50% of patients diagnosed with metastasis to local lymph nodes or distant organs, precluding the possibility of surgical treatment.^72^ ST6Gal1 has been shown to promote epithelial–mesenchymal transition, potentially mediated by α2,6-sialylated EGFR.^73^ Knockdown of ST3Gal3 and ST3Gal4 significantly impairs the binding and rolling of cells facilitated by E-selectin, key events in metastasis.^74^ Additionally, NEU2 expression is down-regulated in human pancreatic cancer tissues. Overexpression of NEU2 inhibits cell migration and invasion, reducing levels of vascular endothelial growth factor, vascular endothelial growth factor receptor, and matrix metalloproteinase 9 (MMP9).^75^ Given the high metastatic potential and treatment challenges associated with PDAC, a deeper understanding of abnormal sialylation offers new insights into potential therapeutic strategies.

Impaired cell death is increasingly recognized as an early event in the development of various cancers.^76^^,^^77^ Aberrant sialylation-induced alterations remodel molecular biological processes, protecting cells from death. The sialyltransferase ST6Gal1 catalyzes the sialylation of tumor necrosis factor receptor 1 (TNFR1), thereby shielding tumor cells from TNF-induced apoptosis.^78^ In hypoxic conditions, ST6Gal1 promotes the accumulation of hypoxia-inducible factor-1 alpha (HIF-1α), further protecting cancer cells from hypoxic stress.^79^ Additionally, the expression of NEU2 is reduced in PDAC cell lines and patient tissues. In NEU2-overexpressing cells, FAS induces extrinsic pathway-mediated apoptosis by catalyzing the desialylation of α2,6-linked sialylation on the FAS receptor.^75^ NEU2 overexpression also decreases sialylation of Sonic Hedgehog, weakening the interaction between Sonic Hedgehog and its receptor Patched1. This suppression of the Hedgehog signaling pathway reduces stemness-like characteristics and enhances apoptosis in pancreatic cancer sphere-forming cells.^80^ Moreover, ST3Gal4 is significantly overexpressed in PDAC. Knockdown of ST3Gal4 reduces the sialylation of proline-rich extensin-like receptor kinase (PERK), activating the PERK–eukaryotic translation initiation factor 2 subunit alpha (eIF2α)–activating transcription factor 4 (ATF4) signaling pathway and shifting PDAC cells from adaptation to cell death due to endoplasmic reticulum stress. ST3Gal4 knockdown also disrupts the interaction between the endoplasmic reticulum and mitochondria, impairing mitochondrial homeostasis and inducing mitochondrial-associated cell death in PDAC.^81^ Targeting sialylation to restore the death sensitivity of cancer cells may present a novel therapeutic strategy for patients with pancreatic cancer.

PDAC is genetically complex and marked by a diverse tumor microenvironment, which plays a significant role in influencing disease prognosis and treatment outcomes. Immunosuppression, however, remains a hallmark characteristic of PDAC.^82^ Siglec-7 and Siglec-9 are key receptors capable of recognizing enhanced sialylation on pancreatic cancer cells. Tumor-expressed sialic acid contributes to the differentiation of monocytes into tumor-associated macrophages via Siglec-7 and Siglec-9 signaling.^83^ Mechanistic studies have shown that α2,3-sialylation activates Siglec-9, which subsequently up-regulates the expression of PD-L1, IL-10, IL-6, and CD206. This activation enhances the immunosuppressive function of tumor-associated macrophages, thereby facilitating immune evasion in PDAC.^83^ Therefore, targeting the sialylation–Siglec signaling axis holds promise as a novel strategy for reversing immune suppression in PDAC.

### Oral cancer

Oral cancer is a major public health issue, with incidence increasing among young adults.^84^ Increased sialylation in patients with oral cancer is associated with disease progression and serves as a potential biomarker for treatment monitoring.^85^ The overexpression of ST3Gal2 and ST3Gal3 contributes to advanced-stage disease, lymph node metastasis, and perineural invasion in oral cancer.^86^ This implies that targeting sialylation has promising clinical value.

### Lung cancer

Lung cancer remains one of the most prevalent and lethal malignancies worldwide. It is primarily classified into non-small cell lung carcinoma (NSCLC) and small cell lung carcinoma, with lung adenocarcinoma being the predominant NSCLC subtype, followed by lung squamous cell carcinoma.^87^^,^^88^ Altered sialylation, particularly α2,6-linked sialylation, has been increasingly recognized as a critical modulator of lung cancer progression.^89^ The sialyltransferase ST6Gal1, a key enzyme catalyzing α2,6-sialylation, exerts multifaceted oncogenic effects. In NSCLC, ST6Gal1 enhances cellular proliferation, migration, and invasion through the neurogenic locus notch homolog protein 1 (Notch1)/MMPs signaling axis.^89^ Its expression is tightly regulated by microRNAs. For example, miR-199a suppresses HER2/HER3 signaling by diminishing ST6Gal1-mediated sialylation of the cell adhesion molecule Nectin-like 2 (Necl-2), thereby attenuating tumor progression (Fig. 6).^90^ Beyond ST6Gal1, other sialyltransferases also constitute a complex regulatory network for the progression of lung cancer. Mutant p53 (R175H) has been shown to promote hepatic metastasis of lung cancer by up-regulating ST6GalNAc1 expression, leading to aberrant sialylation of mucin 5AC (MUC5AC).^91^ Similarly, depletion of ST6GalNAc3 impairs NSCLC cell proliferation, accompanied by altered expression of transferrin receptor protein 1 (TFR1).^92^ Collectively, these findings underscore the central role of dysregulated sialylation in modulating oncogenic signaling and metastatic potential in lung cancer.

Significant progress has been made in the management of NSCLC, yet challenges remain, with drug resistance being the most critical barrier to successful treatment.^93^ Sialylation has been identified as a key factor in the development of drug resistance. For instance, the sialyltransferase ST3Gal4 in NSCLC contributes to acquired resistance to osimertinib, a third-generation EGFR-tyrosine kinase inhibitor, representing a major EGFR-independent resistance pathway.^94^ Moreover, sialylation is also essential in the induction of cisplatin resistance.^95^ Beyond drug resistance, sialylation actively remodels the tumor microenvironment to foster immunosuppression and disease progression. The tumor microenvironment itself is a complex and dynamic ecosystem, comprising a variety of cellular components, including immune cells, cancer-associated fibroblasts, endothelial cells, and pericytes, as well as non-cellular elements such as extracellular matrix components and various signaling molecules.^96^ Sialylation modulates the function of key constituents within this niche. It facilitates extracellular matrix release by cancer-associated fibroblasts, thereby enhancing their pro-tumorigenic potential. In stage I lung adenocarcinoma, patients exhibiting elevated sialylation levels show poorer overall survival. Mechanistically, sialylated sushi domain-containing 2 (SUSD2) interacts with Akt and Smad2, enhancing their phosphorylation and driving extracellular matrix secretion, which collectively amplifies the tumor-promoting activity of cancer-associated fibroblasts.^97^ Furthermore, sialylation directly impairs anti-tumor immunity. Overexpression of ST6GalNAc1 has been observed in aggressive lung adenocarcinoma, where it catalyzes the sialylation of nectin cell adhesion molecule 2 (NECTIN2). Loss of ST6GalNAc1 renders tumor cells more susceptible to T cell-mediated cytotoxicity, emphasizing the role of NECTIN2 sialylation in promoting T cell dysfunction.^98^ Additionally, ST6GalNAc1 evades immune surveillance by promoting ferroptosis in macrophages.^99^ Collectively, these findings not only elucidate how sialylation drives resistance and immunosuppression but also nominate sialyltransferases like ST6GalNAc1 as promising therapeutic targets for NSCLC.

### Renal cell carcinoma

Renal cell carcinoma (RCC) is one of the most prevalent cancers of the urinary system. In recent years, targeted therapies such as sunitinib and immunotherapies, including pembrolizumab, have significantly improved clinical outcomes in RCC patients.^100^ However, challenges such as inconsistent treatment responses and drug resistance remain, highlighting the urgent need for new biomarkers to guide personalized treatment. In this context, sialylation modification has emerged as a promising biomarker with increasing potential value. Clear cell renal cell carcinoma (ccRCC), the most common subtype of RCC, is characterized by elevated levels of sialic acid and fucose compared with healthy renal tissue 101, 102, 103, 104. Increased expression of ST3Gal5 in ccRCC tumors correlates with poor prognosis and immune infiltration, including CD8-positive T cell exhaustion.^105^ Conversely, down-regulation of ST3Gal5 inhibits cell proliferation, migration, and invasion.^106^ ST3Gal1, on the other hand, is down-regulated in ccRCC, and its low expression is associated with a better prognosis.^106^ This may be due to lncRNA maternal expression gene 3 (MEG3), a tumor suppressor, which positively regulates ST3Gal1 mRNA transcription through interaction with c-Jun (Fig. 6).^107^ Overexpression of polysialylated-CD56 enhances ccRCC cell proliferation and invasion through activation of the Hedgehog and Wnt/β-catenin signaling pathways.^108^ Moreover, increased ST6Gal1 expression in ccRCC may serve as an independent risk factor for both survival and recurrence.^109^ In summary, sialylation plays a crucial role in RCC, particularly ccRCC, by regulating key tumor characteristics and influencing prognosis. However, more experimental and clinical studies are needed to better understand its molecular mechanisms and its potential as a therapeutic target or biomarker.

### Bladder cancer

Bladder cancer ranks as the tenth most common malignancy worldwide. Patients with nonmetastatic muscle-invasive bladder cancer have a five-year survival rate of only 36%, which declines to 5% once distant metastasis occurs.^110^, ^111^, ^112^ Understanding the mechanisms driving its metastasis is therefore critical. Up-regulation of ST3Gal6 correlates positively with tumor stage and grade, while its knockout markedly reduces cellular invasion and migration.^113^ The transcription factor GATA binding protein 3 (GATA3), a molecular marker of bladder cancer, negatively regulates ST3Gal6 expression, indicating that the GATA3/ST3Gal6 axis modulates tumor aggressiveness.^113^ Beyond promoting tumor invasion, sialylation also contributes to therapy resistance. It mediates the ubiquitin-dependent degradation of Nectin-4 and suppresses endocytosis of the antibody–drug conjugate enfortumab vedotin (EV). Inhibition of sialyltransferase activity enhances EV-induced immunogenic cell death and augments its anti-tumor efficacy, both as monotherapy and in combination with anti-PD-1 therapy.^114^ Collectively, these findings highlight that targeting sialylation may not only restrain tumor invasiveness but also overcome EV resistance, offering a promising combinatorial strategy for improving outcomes in advanced bladder cancer.

### Prostate cancer

Prostate cancer represents a major global health concern, ranking among the leading causes of cancer-related mortality in men. Its incidence increases significantly with advancing age.^115^ Emerging evidence indicates that altered sialylation contributes to prostate cancer initiation and progression. Elevated ST3Gal1 activity promotes prostate cancer cell proliferation, migration, and tumor growth in xenograft models.^116^ Similarly, ST6Gal1-mediated α2,6-sialylation of N-glycans enhances cell migration and invasion.^117^ Moreover, α2,3-sialylation of the integrin α2 subunit facilitates integrin–ligand interactions that underlie bone metastatic behavior.^118^ Collectively, these findings underscore the pivotal role of sialylation in regulating prostate cancer aggressiveness and metastasis, highlighting the need for further mechanistic studies to define its clinical relevance.

### Breast cancer

Breast cancer remains one of the leading causes of cancer-related mortality among women worldwide, accounting for nearly one-third of all female malignancies, and with its mortality rate constituting about 15% of the total number of cases diagnosed. Its incidence and progression are influenced by a complex interplay of genetic, environmental, and lifestyle factors.^119^^,^^120^ Increasing evidence indicates that aberrant sialylation contributes to breast cancer development and progression. Compared with adjacent normal tissues or normal breast epithelial cells, breast cancer tissues and MDA-MB-231 cells exhibit elevated levels of N-linked sialylated glycans.^121^ ST3Gal1 facilitates cell proliferation and survival in MCF7 cells by enhancing glial cell line-derived neurotrophic factor (GDNF)-mediated rearranged during transfection (RET) signaling. Mechanistically, GDNF activates the PI3K/Akt pathway, leading to phosphorylation of specific protein 1 (Sp1), which binds to the ST3Gal1 promoter and up-regulates its expression.^122^ In addition, silencing of ST8Sia4 significantly inhibits the proliferation and invasion of MDA-MB-231 cells both *in vitro* and *in vivo*. Notably, miR-26a and miR-26b negatively regulate ST8Sia4, revealing a post-transcriptional mechanism by which microRNAs modulate sialylation and influence breast cancer aggressiveness (Fig. 6).^121^ Collectively, these findings highlight the multifaceted role of sialylation in sustaining the malignant phenotype of breast cancer. Targeting this regulatory network, such as microRNA-based strategies, may represent a novel therapeutic approach for breast cancer management.

Despite advances in surgery, radiotherapy, chemotherapy, and targeted therapies, drug resistance remains a major obstacle in breast cancer treatment. Understanding the molecular basis of resistance and identifying predictive or reversible mechanisms are essential for improving patient outcomes.^123^ Sialylation has recently been implicated in mediating therapeutic resistance in breast cancer. ST3Gal4 catalyzes α2,3-sialylation of HSP90B1, promoting its retrograde trafficking from the Golgi to the endoplasmic reticulum. Endoplasmic reticulum-localized HSP90B1 enhances the clearance of radiotherapy-induced misfolded proteins and activates the PERK/eIF2α/ATF4 pathway, which suppresses radiotherapy-induced reactive oxygen species accumulation, thereby driving radiotherapy resistance and poor clinical outcomes.^124^ In addition, ST3Gal1-mediated sialylation of neuropilin-1 (NRP1) amplifies EGF/EGFR downstream signaling, increasing tumor cell motility and conferring resistance to cetuximab, whereas ST3Gal1 silencing sensitizes cells to cetuximab-mediated cytotoxicity.^125^ Another mechanism involves SIA-IgG, which carries a unique sialylated N-glycan at site 162 of the IgG heavy chain. SIA-IgG promotes glycolysis and lactate recycling, driving metabolic reprogramming that supports breast cancer progression. This makes SIA-IgG a promising target for targeted therapy in breast cancer.^126^ These findings indicate that aberrant sialylation promotes resistance to both radiotherapy and targeted therapies while facilitating metabolic adaptation. Targeting specific sialyltransferases may therefore represent a promising strategy to overcome therapy resistance and reprogram tumor metabolism, ultimately improving treatment responses in patients with breast cancer.

Breast cancer displays a distinct metastatic tropism, with the bone, liver, lung, and brain being the most frequent metastatic sites, and metastatic breast cancer remains associated with a dismal prognosis.^127^^,^^128^ α2,6-sialylation has emerged as a critical regulator of metastatic behavior. ST6Gal1 drives α2,6-sialylation of platelet/endothelial cell adhesion molecule 1 (PECAM-1), facilitating transendothelial migration through the pulmonary vasculature, a crucial step in lung metastasis. Silencing either ST6Gal1 or PECAM-1 markedly reduces lung colonization.^129^ However, the role of ST6Gal1 appears to be context-dependent: its loss can enhance circulating tumor cell aggregation and promote lung colonization, whereas its overexpression suppresses dissemination. Podocalyxin-like protein 1 (PODXL), an ST6Gal1 substrate, contributes to the formation of dormant circulating tumor cell clusters, linking sialylation to metastatic dormancy.^130^ Furthermore, α2,3-sialylation modulates Galectin-8-mediated adhesion in MDA-MB-231 cells, further influencing metastatic interactions with the extracellular matrix.^131^ Brain metastasis, another clinically significant manifestation of advanced breast cancer, is strongly linked to aberrant sialylation. ST6GalNAc5 promotes breast cancer cell adhesion to the blood–brain barrier, facilitating brain colonization.^132^ Elevated expression of the circular RNA circNAV3 (hsa_circ_0008509) promotes brain metastasis by sponging miR-4262, thereby up-regulating ST6GalNAc5 and enhancing EGFR signaling through sialylation.^133^ Consistently, highly sialylated MDA-MB-231BR cells exhibit increased invasiveness and blood–brain barrier penetration capacity.^134^^,^^135^ Although the precise mechanisms governing blood–brain barrier transmigration remain incompletely understood, current evidence underscores sialylation as a pivotal molecular determinant of organ-specific metastasis in breast cancer.

### Ovarian cancer

Ovarian cancer ranks as the eighth most common malignancy among women globally.^136^^,^^137^ Ovarian cancer is initially highly responsive to platinum-based chemotherapy. However, most patients experience relapse after primary surgery and chemotherapy, underscoring the urgent need for novel therapeutic strategies to improve long-term outcomes.^138^ Among its molecular alterations, aberrant sialylation, particularly mediated by sialyltransferases such as ST6Gal1 and ST3Gal1, plays a pivotal role in tumor progression. ST6Gal1 expression is up-regulated in all major subtypes of ovarian cancer.^139^ Its overexpression confers cancer stem cell-like properties and enhances tumorigenicity *in vivo*.^140^ Mechanistically, Sox2 up-regulates ST6Gal1 and α2,6-sialylation, indicating that the stemness-associated phenotype driven by Sox2 is at least partly mediated through ST6Gal1 activity.^141^ Moreover, ST6Gal1 promotes α2,6-sialylation of fibroblast growth factor receptor 1 (FGFR1), facilitating cell migration, and regulates HIF-1 to support tumor survival under hypoxic conditions.^79^^,^^142^^,^^143^ Elevated ST6Gal1 expression further enhances cellular invasiveness, while sustained EGFR signaling, maintained by ST6Gal1-induced EGFR membrane retention, drives additional motility and metastatic potential.^144^ ST3Gal1 is likewise up-regulated in ovarian cancer, promoting cell proliferation, migration, and peritoneal dissemination through EGFR signaling.^145^^,^^146^ The transcription factor GATA2 also contributes to cell migration by regulating ST3Gal4-mediated sialylation.^147^ Together, these findings define sialylation as a critical regulator of ovarian cancer aggressiveness, supporting proliferation, invasion, and metastatic dissemination.

Metabolic reprogramming is a hallmark of cancer, and sialylation has been found to be a key molecule connecting this process. ST6Gal1 enhances glycolytic flux by activating phosphofructokinase and hexokinase, two key glycolytic enzymes, thus enabling cancer cells to gain metabolic adaptive advantages.^143^ In addition to regulating cellular metabolism, sialylation also actively participates in the immunosuppressive remodeling of the tumor microenvironment. In high-grade serous ovarian cancer, silencing ST3Gal3 suppresses tumor growth and reprograms tumor-associated macrophages from an M2-like pro-tumor phenotype to an M1-like tumor-suppressive state, suggesting that modulation of sialylation may reshape the immunosuppressive tumor microenvironment and enhance the efficacy of immunotherapy.^148^

Platinum-based chemotherapy regimens remain the standard first-line treatment for ovarian cancer. Forced ST6Gal1 expression reduces caspase-3 activation and enhances cell survival following cisplatin treatment, implicating ST6Gal1-mediated inhibition of apoptosis in cisplatin resistance.^149^ In addition to resistance to platinum-based drugs, elevated ST6Gal1 expression also contributes to resistance to several other chemotherapeutic agents, including paclitaxel, Adriamycin, and gemcitabine.^150^ Similarly, increased ST3Gal1 expression correlates with paclitaxel resistance.^145^ The suppression of ST3Gal3 enhances the activation of caspase-8/3 signaling pathways in response to paclitaxel treatment.^151^ Collectively, these studies reveal that sialylation reprograms key oncogenic processes, including stemness, metabolism, immune evasion, and drug response, underscoring its potential as a therapeutic target in ovarian cancer.

### Cervical cancer

Cervical cancer is the fourth most common cancer after breast cancer, CRC, and lung cancer in women worldwide.^152^^,^^153^ Researchers have noted that the intensity and distribution of α2,3-linked sialic acid and α2,6-linked sialic acid increase as the squamous intraepithelial lesion escalates in severity. These sialylation changes might be crucial for cellular transformation before tumorigenesis.^154^ HeLa cells are more sensitive to cisplatin when ST6Gal1 is knocked down.^155^ However, ST3Gal4 might function as a tumor suppressor in cervical cancer. There is an inverse association between ST3Gal4 expression and the pathological grading of cervical cancer tissues. Elevated ST3Gal4 levels have been shown to curtail the growth and proliferation of cervical cancer cells both *in vivo* and *in vitro*, exerting its effects through the Notch1/p21/cyclin-dependent kinases (CDKs) signaling pathway.^156^ Taken together, sialylation may have a vital part to play in cervical cancer.

## Sialylation in hematological malignancies

Hematological malignancies, characterized by their high incidence and heterogeneity, have been studied for the involvement of sialylation in their pathogenesis. Research has demonstrated that aberrant sialylation contributes to the progression of various hematological malignancies, including lymphoma, leukemia, and plasma cell dyscrasias such as multiple myeloma.

### Leukemia

Acute lymphoblastic leukemia is the most prevalent type of hematological malignancy in children. In childhood acute lymphoblastic leukemia, the up-regulation of ST6Gal1 and ST3Gal5 expression has been observed in lymphoblasts. Moreover, the levels of ST6Gal1 and ST3Gal5 are positively correlated with the higher risk of acute lymphoblastic leukemia in children. However, the expression of ST8Sia1 is down-regulated in patients, suggesting differential regulation of sialyltransferases during leukemogenesis.^157^

Despite significant advances in understanding the genetics of leukemia and the development of targeted and personalized therapies, major challenges remain, particularly those related to drug resistance. Aberrant sialylation has emerged as a key molecular mechanism driving drug resistance across various types of leukemia. In acute myeloid leukemia, sialylation plays a regulatory role in the activity of the PI3K/Akt signaling pathway, P-glycoprotein (P-gp), and multidrug resistance-related protein 1 expression, potentially leading to the development of multidrug resistance.^158^ In chronic myeloid leukemia, ST8Sia4 controls the level of P-gp expression and the activity of PI3K/Akt signaling pathway, thus contributing to the multidrug resistance of chronic myeloid leukemia cells.^159^ In addition, post-transcriptional regulation also plays a crucial role in sialylation-mediated drug resistance. miR-4701-5p has the ability to directly target ST3Gal1, reduce the resistance to chemotherapeutic drugs *in vitro*, and transform adriamycin-resistant to sensitive tumor cells *in vivo*.^160^ ST3Gal4 expression in the imatinib-resistant cells increases significantly. Further studies discovered that miR-224 and hsa-let-7i (let-7i) could regulate ST3Gal4 expression, which in turn controls cell proliferation and chemosensitivity.^161^ Additionally, imatinib mesylate resistance in chronic myeloid leukemia is strongly correlated with the changed levels of total sialic acid, ST3Gal1, and ST3Gal2.^162^ In addition to its direct involvement in drug resistance mechanisms, sialylation also modulates the immune recognition of leukemia cells. Together with impaired natural killer (NK) response, sialylation of chronic lymphocytic leukemia cells could promote immune escape. And the sialylation of CD49d could regulate vascular cell adhesion molecule 1-dependent and fibronectin-dependent migration.^163^ Targeting key sialyltransferases or their regulatory networks may therefore offer new opportunities to overcome resistance and improve therapeutic outcomes.

### Others

Recent studies have revealed that sialylation facilitates the dissemination of lymphoma cells from the primary tumor site into the bloodstream. Knockdown of ST6Gal1 expression or enzymatic desialylation by neuraminidase enhances cell adhesion, thereby impeding lymphoma cell invasion and metastasis.^164^ Furthermore, sialylation appears to protect lymphoma cells from apoptosis induced by intravascular stress. In multiple myeloma, ST3Gal6 has been identified as a critical determinant of bone marrow homing and therapeutic resistance.^165^ These findings highlight the crucial role of sialylation in regulating hematologic malignancy progression.

## Detection methods of sialylation

To accurately measure sialic acid expression levels and spatial distribution, current detection methods are generally divided into *in vitro* and *in situ* methods. While *in vitro* detection has been the dominant technique for sialic acid analysis due to its simplicity and reproducibility, recent advances in glycomics and imaging technologies have highlighted the importance of *in situ* detection methods, which preserve the original structure and spatial information of glycan chains.^166^^,^^167^

### In vitro detection

For *in vitro* detection, sialic acid is typically released from glycan chains via neuraminidase or acid hydrolysis.^168^^,^^169^ Following this, purification is commonly achieved using anion exchange columns or borate affinity columns. These column-based purification methods have been widely employed since the mid-20th century.^170^ Despite their relatively low throughput, these techniques remain invaluable for analyzing complex biological samples, offering significant utility in the qualitative analysis and preliminary quantification of sialic acid.

Among the earliest methods, colorimetric assays, such as the use of Bial's reagent and Warren's thiobarbituric acid assay, were widely employed to detect sialic acid through the formation of colored products.^170^ The colorimetric method could be completed quickly, although it is not particularly sensitive or specific.^171^ A colorimetric sensor, a promising method for rapidly screening diseases marked by sialic acid, has been developed with the advancement of biosensor technology.^172^ The fluorometric method for sialic acid detection usually involves the reaction of sialic acid with oxidants or acids. Fluorometric methods, including those utilizing 3,5-diaminobenzoic acid, offer enhanced sensitivity, enabling the detection of low levels of sialic acid.^171^ Additionally, chromatographic techniques, particularly high-performance liquid chromatography coupled with fluorescence detection or mass spectrometry, have become essential tools for the detailed analysis of sialylation, allowing for precise quantification and structural characterization of sialylated glycoproteins.^171^^,^^173^^,^^174^ However, these *in vitro* approaches, while providing valuable insights, are limited by their inability to reflect the complex, dynamic nature of sialylation in living organisms.

### *In situ* detection

*In situ* detection allows for the retention of the spatial distribution of sialylated glycans on cell membranes or within tissue architecture, thus providing crucial insights into the biological functions of sialylation in its native context. However, detecting sialylation remains challenging due to several factors.

Glycans, owing to their conserved sequences and structures, exhibit low immunogenicity, making it difficult to induce robust T cell-dependent immune responses.^166^^,^^167^ As a result, only low-affinity, low-specificity IgM antibodies are typically generated.^175^ Furthermore, antibodies developed using glycopeptides as antigens tend to recognize the entire glycopeptide structure rather than the glycan itself, limiting their specificity to glycan modifications.^176^^,^^177^ Antibodies specific to sialylated glycan epitopes are primarily restricted to a few well-characterized targets, including polysialic acid, sialyl-Lewis x, and sialyl-Tn.^178^, ^179^, ^180^ Although several commercial antibodies for pan-specific sialylated glycans are available, their affinity and specificity have not been systematically validated, limiting their application in complex biological samples.^177^

In contrast, lectins, as natural glycan-binding proteins, have been extensively used for the detection of sialylated glycans.^167^^,^^181^ Similar to antibodies, lectins can be directly applied to techniques such as Western blotting, enzyme-linked immunosorbent assay, flow cytometry, and confocal microscopy without the need for prior sialic acid release. For example, Sambucus nigra lectin (SNA) specifically recognizes α2,6-linked sialic acid, while Maackia amurensis lectin II (MAL-II) recognizes the α2,3-linked form, both of which have been widely used to analyze glycan linkage types on cell membranes.^177^ However, lectins also have limitations, including low substrate specificity and non-specific binding, which make it difficult to distinguish between different sialylation modifications in complex samples.^177^^,^^182^ To overcome these issues, a novel *in situ* detection platform based on engineered glycosidases, glycan recombinant affinity binders (GRABs), has been developed. GRABs, as a class of multifunctional sialoglycan-binding agents, exhibit high specificity and affinity for sialoglycan substrates.^177^

Additionally, surface-enhanced Raman spectroscopy offers both *in vitro* and *in situ* detection capabilities, with high sensitivity, high resolution, and non-destructive characteristics, making it an invaluable tool for the study of glycan structures.^183^ The application of surface-enhanced Raman spectroscopy in the detection of sialylation levels has been comprehensively reviewed, highlighting its substantial contributions to the advancement of glycomics research.^184^

Overall, with the development of high-resolution imaging and protein engineering, *in situ* detection methods are gradually becoming the new trend in sialic acid research. Future efforts will focus on improving the specificity, throughput, and ease of use of these methods, which will provide more powerful tools for studying the role of sialic acid in development, cancer, and immune-related diseases.

## Diagnostic and prognostic value of sialylation

### As biomarkers in malignancies

Therapeutic and diagnostic strategies targeting sialylation have been the subject of many studies. About 90% of human cancers express immunoreactive Thomsen-Friedenreich (TF) sugar antigens, but not in healthy tissues. And naturally occurring TF-specific antibodies are linked to tumor progression.^185^^,^^186^ The abnormal sialylation of TF specific antibody may serve as a promising serological biomarker of gastric cancer.^187^^,^^188^ In a similar context, studies have shown that the low sialylation and high avidity of TF-specific IgA antibodies exhibit the most robust diagnostic potential in patients with breast cancer, with a diagnostic accuracy of about 80%.^189^ Additionally, serum sialic acid level is considered a promising biomarker for the diagnosis and prognosis of prostate cancer.^190^ Prostate-serum specific antigen (PSA), a protein produced by prostate cells, serves as the main biomarker for the detection of prostate cancer. Increased α2,3-sialylation of PSA may be associated with prostate cancer progression.^191^ The expression of α2,3-sialylated PSA (S23PSA) in Gleason 4 and 5 prostate tissues is higher than in benign prostate tissues. And the diagnostic performance of S23PSA density is better than that of routine examination.^192^ The α2,3-Sia-PSA/α1,6-Fuc-PSA (SF index) could distinguish high-grade prostate cancer and provide helpful information for prostate biopsy decision-making in men who have an abnormal PSA level.^193^ However, unlike benign prostatic hyperplasia and indolent prostate cancer, the percentage of α2,6-sialylated PSA in aggressive prostate cancer is significantly lower.^194^ In addition, the levels of HER2 sialylation and the HER2 mutation may serve as reliable biomarkers for gastric cancer anti-HER2 treatment.^36^^,^^38^ The above studies have demonstrated that sialylation can be used as an effective biological marker for disease progression.

### Prognostic value in malignancies

In recent years, novel systemic treatments have been making strides in the treatment of tumors. This includes promising new therapeutic strategies such as immune checkpoint inhibitors, targeted therapies, and combination regimens.^33^^,^^43^^,^^63^^,^^120^^,^^195^ Studies have demonstrated that understanding the molecular and immune profiles of tumors can improve the precision of these treatments.^196^^,^^197^ As therapeutic options continue to evolve, the integration of biomarkers such as sialylation offers a new layer of precision in predicting treatment responses and guiding personalized treatment strategies. Tumor-infiltrating lymphocytes are heterogeneous lymphocyte subsets that play an important role in chemotherapy.^198^^,^^199^ Sialylation could impact the treatment response and prognosis by regulating tumor-infiltrating lymphocytes in breast cancer patients.^200^ Moreover, a study demonstrates that a low level of α2-3-sialylation may improve the efficacy of chemotherapy.^201^ In invasive chemotherapy-resistant breast cancer, uncontrolled sialylation led to an excessive negative charge on the cell membrane and stimulated the repulsive force between tumor cells. This means early metastasis, rapid disease progression, and fatal consequences.^202^ Moreover, it is possible to use sialylation level as a potential biomarker for predicting the effectiveness of JAK or MEK inhibitors in the treatment of acute myeloid leukemia.^203^ Wu et al proposed that the increased sialylation of IgG could be a biomarker to evaluate the efficacy of anti-PD-1(L1) therapy in HCC.^204^ This has significant implications for the immune checkpoint therapy development.

## Therapeutic strategies

Cell surface sialosides constitute a central axis of immune modulation that is exploited by tumors to evade both innate and adaptive immune destruction. Therapeutic strategies that target tumor-associated sialosides may therefore potentiate antitumor immunity.^205^

### Sialyltransferases inhibitors

#### *P-3FAX-Neu5Ac*

P-3FAX-Neu5Ac inhibits sialyltransferase enzymes, which add Neu5Ac to glycoproteins and glycolipids on cell surfaces. Intracellularly, P-3FAX-Neu5Ac, which is incapable of being utilized as a substrate by biosynthetic enzymes, is converted into the active inhibitor CMP-3FAX-Neu5Ac. This leads to reduced sialylation globally by inhibiting all sialyltransferases. However, P-3FAX-Neu5Ac has been associated with liver and kidney dysfunction in murine models.^206^ For prospective therapeutic strategies in cancer, researchers are looking into novel compounds like C-5-modified 3-fluoro sialic acid sialyltransferase inhibitors and better-tolerated versions of P-3FAX-Neu5Ac.^207^

#### *Lithocholic acid and its analogues*

A lithocholic acid named soyasaponin I could decrease the α2,3-sialylation and increase E-cadherin expression, thus inhibiting cell migration. On this basis, the lithocholic acid analogue AL10, which exhibits cellular permeability, effectively reduces sialylation on the cell surface. Notably, AL10 does not impact liver and renal function in experimental animals, but it significantly inhibits experimental lung metastases *in vivo*.^208^ Lith-O-Asp, another lithocholic acid analogue, is a novel sialyltransferase inhibitor with promising antimetastatic and antiangiogenic effects.^209^ By lithocholic acid extension strategy, researchers have designed and synthesized Lith-O-Asp analogue, FCW34 and FCW66, which are potential sialyltransferase inhibitors and inhibit cell migration in MDA-MB-231 cells. In animal models, FCW34 could exert anti-tumor effects by inhibiting tumor growth, reducing angiogenesis, and delaying cancer cell metastasis.^210^

#### *Other sialyltransferase inhibitors*

Natural medicine has garnered substantial attention for its unique advantages in the treatment of tumors. Ginsenosides, the principal active ingredient of ginseng, have been extensively studied, with approximately 200 ginsenosides identified to date.^211^ Studies have shown that four ginsenosides, including 20(S)-ginsenosides Rg3, 20(R)-ginsenosides Rg3, 20(S)ginsenosides Rh2, and 20(R)-ginsenosides Rh2, inhibit the expression of sialyltransferases in a dose-dependent manner.^212^ Moreover, challenges remain in developing subtype-selective and highly bioavailable sialyltransferase inhibitors.

### Antibodies targeting Siglecs

By interacting with sialylated glycans that are frequently overexpressed on the surface of tumor cells, inhibitory Siglecs transmit suppressive signals that attenuate immune cell activation, thereby promoting immune evasion and tumor progression. To counteract this immunosuppressive mechanism, various therapeutic strategies have been developed to block Siglec-mediated signaling pathways.^213^ While previous reviews have systematically summarized the Siglec family,^213^^,^^214^ this section focuses specifically on the development and therapeutic potential of antibodies targeting Siglecs in the context of cancer immunotherapy.

Siglec-6 is an inhibitory receptor that has been explored as a target for cancer immunotherapy. Cyr et al identified high-affinity, patient-derived anti-Siglec-6 monoclonal antibodies, RC-1 and RC-2, which were subsequently engineered into T cell-engaging bispecific antibodies (T-biAbs). These T-biAbs are capable of simultaneously binding to Siglec-6 and CD3, thereby facilitating T cell recruitment to malignant cells and promoting T cell-mediated cytotoxicity against chronic lymphocytic leukemia cells.^215^

Siglec-9 shares approximately 84% sequence homology with Siglec-7.^216^ The development of anti-Siglec-7 monoclonal antibodies has been shown to activate NK cells and induce potent antitumor immunity against ovarian cancer.^217^ In parallel, Choi et al developed a highly specific monoclonal antibody (8A1E9) targeting Siglec-9, which demonstrated significant anti-tumor activity both *in vitro* and *in vivo* in ovarian cancer models.^218^^,^^219^ Furthermore, Fab fragments targeting Siglec-7 and Siglec-9 have been shown to significantly enhance NK cell-mediated cytotoxicity by blocking ligand–receptor interactions.^220^ Collectively, these findings highlight the therapeutic potential of Siglec-7 and Siglec-9 blockade as innovative immunotherapeutic strategies.

Siglec-15 has recently been identified as a critical immune suppressor in the tumor microenvironment. Antibodies against Siglec-15 are being actively investigated to overcome immune resistance and restore antitumor immunity. NC318 has entered phase II clinical trials (NCT04699123) and demonstrated preliminary efficacy in patients with NSCLC who are refractory to PD-1 blockade.^221^ In preclinical studies, several additional antibodies, including 3F1 and 1-15D1, have exhibited potent antitumor activity.^222^^,^^223^ Furthermore, S15-4E6A has demonstrated notable antitumor activity against Siglec-15-positive lung adenocarcinoma by modulating the polarization of macrophages.^224^ Collectively, these findings underscore the potential of Siglec-15-targeted antibodies as novel immunotherapeutic agents.

### Sialidase-fused BiTEs and CiTEs

Bispecific T cell engagers (BiTEs), a subclass of bispecific antibodies, function by simultaneously binding a tumor-associated antigen and T-cell receptors, thereby redirecting T cells to lyse tumor cells. To enhance their efficacy in solid tumors, BiTEs have been engineered as fusion proteins with sialidases, enabling localized desialylation at the immunological synapse. These BiTE–sialidase fusion proteins have demonstrated superior T cell activation, immunological synapse formation, and tumor cell lysis compared with parental BiTEs in both xenograft and syngeneic mouse models.^225^ Moreover, checkpoint inhibitory T cell engagers (CiTEs), which incorporate immune checkpoint blockade into BiTE scaffolds, exhibit enhanced therapeutic efficacy. Inclusion of sialidase within CiTE constructs significantly potentiates T cell-mediated cytotoxicity *in vitro*, highlighting the synergistic effect of glycan editing and checkpoint inhibition.^226^

### Antibody–sialidase conjugates

Antibody–sialidase conjugates represent a precise glycoengineering strategy for enhancing antitumor immunity. By fusing recombinant sialidases to tumor-targeting antibodies such as HER2-specific antibodies, these constructs selectively desialylate the tumor cell glycocalyx in an antigen-dependent manner. This desialylation reduces engagement with inhibitory Siglec receptors on NK cells while enhancing activation via NK receptors like NKG2D, thereby boosting antibody-dependent cellular cytotoxicity.^205^ In breast cancer models, antibody-sialidase treatment increased immune cell infiltration and activation, significantly prolonging survival. This immune enhancement was shown to depend on Siglec-E expression on tumor-infiltrating myeloid cells.^227^ Notably, the trastuzumab–sialidase conjugate E−301 remodeled the tumor microenvironment, promoting a shift toward antitumoral macrophage populations and augmenting CD8-positive T cell responses.^228^

### NK cell surface glycoengineering

Tumors often suppress NK cell activation by engaging Siglec-7 through their hypersialylated surface glycans. To counteract this, sialidase was assembled onto the NK cell surface using DNA-mediated protein anchoring technology, generating sialidase-displaying NK cells. This strategy effectively removes immunosuppressive sialic acid at the NK-tumor cell immunological synapse, unleashing NK cell effector functions and enhancing cytotoxic responses.^229^ Chemoenzymatic glycan editing has been employed to decorate NK cells with high-affinity CD22 ligands, also known as Siglec-2, enabling the selective recognition and lysis of malignant B cells while sparing healthy B cells. Further functionalization with E-selectin ligands facilitates efficient homing of these engineered immune cells to the bone marrow, thereby enhancing therapeutic efficacy.^230^

### Others

A novel glycoengineering strategy employs a penta-functional dendritic probe (Den@5F) to simultaneously block tumor cell surface sialic acid and deliver galactose residues. This hypergalactosylation promotes immune recognition and cytotoxicity by favoring glycan patterns that stimulate immune activation. *In vivo*, this approach significantly enhances anti-tumor immune responses, suggesting a potent glycan-directed strategy for immunomodulation.^231^ Additionally, sialidases are bioorthogonally decorated onto the surface of azido-functionalized bioengineered bacteria for recognizing tumor sialoglycans and cleaving their sialosides into free sialic acid. Sialidase-mediated cleavage of tumor cell sialoglycans releases free sialic acids that activate the bacterial gene circuit to induce hemolysin E expression, leading to tumor cell lysis. Meanwhile, tumor cell desialylation reverses the immunosuppressive effect of glycoimmune checkpoints and further improves the therapeutic effect of solid tumors.^232^

## Conclusion

Sialylation plays a pivotal role under both physiological and pathological conditions, with its levels primarily regulated by the activity of sialyltransferases and NEUs. Dysregulation of these enzymes is commonly observed in human malignancies, leading to aberrant sialylation. An increasing body of research has highlighted the significance of abnormal sialylation in the development, treatment, and prognosis of tumors. These studies have begun to elucidate the intricate molecular mechanisms governing the regulation of sialyltransferases and NEUs, including the impact of non-coding RNAs, which are known to influence their expression and activity (Fig. 6). Understanding these mechanisms is vital for developing targeted therapies and improving clinical outcomes for cancer patients.

In most tumors, up-regulated sialylation could make tumor cells sustain proliferation, increase adhesion and invasion, evade cell death, and promote drug resistance. However, Zhou et al propose that ST6Gal1 expression, which is negatively correlated with the metastatic potential, may have a role in tumor suppression in HCC. This is inconsistent with our notion that sialylation promotes tumor progression. Thus, the role of sialylation in tumor progression should be further explored. Sialyltransferase inhibitors, anti-Siglecs antibodies, and antibody–sialidase conjugates are the promising therapeutics.^12^ Notably, hypersialylation may play a cytoprotective role, and treatments aimed at eliminating tumor-associated sialylation should be used cautiously.^233^ In addition, blocking the sialylation of IgG *in vivo* could enhance anti-PD-1(L1) therapy-induced antitumorigenic immunity in HCC. This has an essential contribution to the combination of immune checkpoint therapy and sialylation-targeted therapy.

To date, more efforts are still required to elucidate the novel roles and mechanisms of sialylation. The mechanisms of sialylation in some cancers, including gallbladder cancer, cervical cancer, and lymphoma, are still insufficient. Moreover, research and development of sialyltransferase inhibitors also confront various challenges, including subtype selectivity, cell permeability, and bioavailability. Addressing these challenges is crucial for the advancement of potential therapeutic strategies against human malignancies, where sialylation plays a significant role.

## CRediT authorship contribution statement

**Ran Kong:** Writing – review & editing, Writing – original draft, Investigation. **Cong Wang:** Writing – review & editing, Writing – original draft. **Yu Zhang:** Writing – review & editing. **Guangcai Zhong:** Writing – review & editing. **Jiarui Liu:** Writing – review & editing, Validation, Supervision, Funding acquisition. **Xiangxiang Zhou:** Writing – review & editing, Writing – original draft, Validation, Supervision, Investigation, Funding acquisition.

## Funding

This work was supported by the 10.13039/501100001809National Natural Science Foundation of China (No. 82570247, 82170189), the 10.13039/501100007129Shandong Provincial Natural Science Foundation (No. ZR2021YQ51, ZR2022QH204), and Taishan Scholars Program of Shandong Province, China (No. tstp20250756).

## Conflict of interests

The authors declared no conflict of interests.
